# Perfect Spherical Tetrahedral Metallo-Borospherene Ta_4_B_18_ as a Superatom Following the 18-Electron Rule

**DOI:** 10.1021/acsomega.1c00828

**Published:** 2021-04-12

**Authors:** Yu Zhang, Xiao-Qin Lu, Miao Yan, Si-Dian Li

**Affiliations:** Institute of Molecular Science, Shanxi University, Taiyuan 030006, China

## Abstract

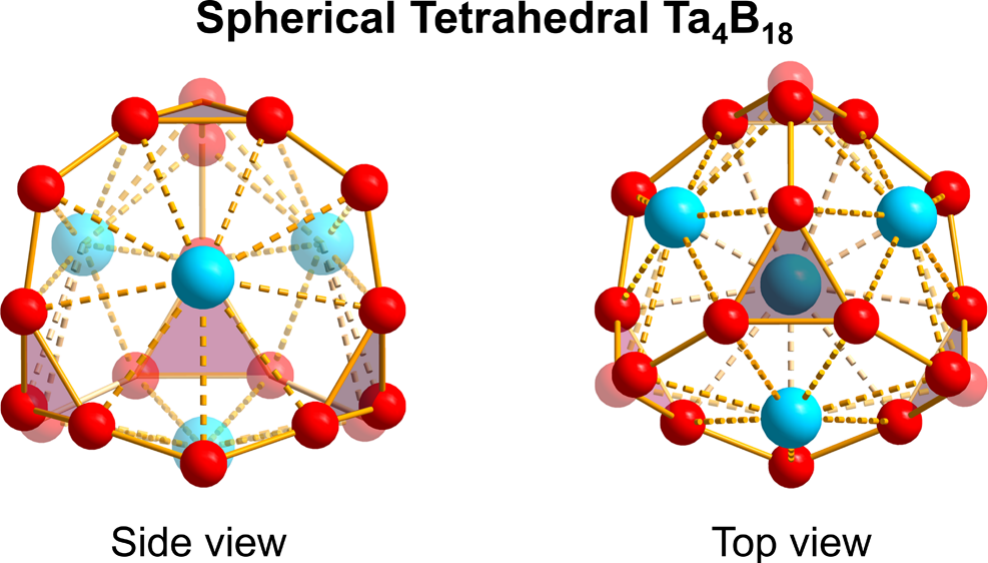

Cage-like metallo-borospherenes exhibit unique structures and bonding. Inspired by the newly reported smallest spherical trihedral metallo-borospherene *D*_3h_ Ta_3_B_12_^–^ (**1**), which contains two equivalent B_3_ triangles interconnected by three B_2_ units on the cage surface, we present herein a first-principles theory prediction of the perfect spherical tetrahedral metallo-borospherene *T*_d_ Ta_4_B_18_ (**2**), which possesses four equivalent B_3_ triangles interconnected by six B atoms, with four equivalent nonacoordinate Ta centers in four η^9^-B_9_ rings as integrated parts of the cage surface. As the well-defined global minimum of the neutral, Ta_4_B_18_ (**2**) possesses four 10c-2e B_9_(π)–Ta(d_σ_) and eight 10c-2e B_9_(π)–Ta(d_δ_) coordination bonds evenly distributed over four Ta-centered Ta@B_9_ nonagons, with the remaining 18 valence electrons in nine 22c-2e totally delocalized bonds following the 18-electron principle (1S^2^1P^6^1D^10^) of a superatom. Such a bonding pattern renders spherical aromaticity to the tetrahedral complex, which can be used as building blocks to form the face-centered cubic crystal Ta_4_B_15_ (**3**). The IR, Raman, and UV–vis spectra of Ta_4_B_18_ (**2**) are theoretically simulated to facilitate its future experimental characterizations.

## Introduction

Boron as a prototypical electron-deficient element exhibits unique structures and bonding in bulk allotropes, polyhedral molecules, and gas-phase clusters.^1−3^ Combined photoelectron spectroscopy (PES) and first-principles theory investigations in the past two decades have unveiled a rich landscape for size-selected boron clusters (B*_n_*^–/0^) from planar or quasi-planar species (*n* = 3–38, 41, and 42) to cage-like borospherenes (*C*_3_/*C*_2_ B_39_^–^ and *D*_2d_ B_40_^–/0^) featuring delocalized multicenter two-electron (mc-2e) σ and π bonds, with B_39_^–^ being the only boron cluster monoanion possessing a cage-like global minimum (GM).^2−9^ Seashell-like *C*_2_ B_28_^–/0^ and *C*_s_ B_29_^–^ were later observed in PES measurements as minor isomers coexisting with their quasi-planar GM counterparts.^7,8^ Endohedral M@B_40_ (Ca, Sr, Sc, Y, and La) and exohedral M&B_40_ (M = Be and Mg) metallo-borospherenes were proposed in theory shortly after the discovery of *D*_2d_ B_40_^–/0^.^10,11^ Endohedral *D*_2_ Ta@B_22_^–^ and *D*_2d_ U@B_40_ were predicted to be superatoms following the 18-electron rule and 32-electron principle, respectively.^12,13^ Other cage-like B*_n_* clusters (*n* = 20, 30, 38, 40, 50, and 60) and related Ti-doped species have also been predicted in theory.^14,15^ Joint ion-mobility measurements and density functional theory (DFT) investigations indicated that B*_n_*^+^ boron cluster monocations possess double-ring tubular structures in the size range between *n* = 16 and 25.^16^ Extensive GM searches and DFT calculations showed that B_46_ is the smallest core–shell boron cluster with a B_4_ core at the center (B_4_@B_42_), while B_48_, B_54_, B_60_, and B_62_ are the first bilayer boron clusters predicted to date.^17,18^ Encouragingly, bilayer B_48_^–/0^ has been very recently confirmed in gas-phase PES measurements, revealing a new structural domain in boron nanoclusters and nanomaterials.^19^

Transition metal-doping generates interesting structures and bonding in boron clusters. Typical examples include the experimentally confirmed monometal-centered boron wheels M@B*_n_* (Co@B_8_^–^, Ru@B_9_^–^, and Ta@B_10_^–^), double-ring tubular boron drums M@B*_n_*^–^ (Mn@B_16_^–^, Co@B_16_^–^, Rh@B_18_^–^, and Ta@B_20_^–^), and di-metal-doped inverse-sandwich *D*_6h_ Ta_2_&B_6_^–/0^.^20−26^ Di-La-doped inverse-sandwich-type mono-decker La_2_B*_n_*^–^ (*n* = 7–9)^27,28^ and tri-La-doped inverse triple-decker La_3_B_14_^–^ were also observed in PES experiments.^29^ Double-ring tubular *C*_2h_ La_2_B_20_ (La_2_[B_2_@B_18_) was predicted to be a molecular rotor with fluxional bonds at room temperature.^30^ The first tri-La-doped spherical trihedral metallo-borospherene *D*_3h_ La_3_B_18_^–^ with three decacoordinate La centers as integral parts of the cage surface has been discovered recently in a combined experimental and theoretical investigation.^31^ The first core–shell spherical trihedral metallo-borospherene *D*_3h_ La_3_B_20_^–^ (La_3_[B_2_@B_18_]^−^) with a B_2_ core was predicted shortly after.^32^ The smallest tri-Ta-doped spherical trihedral metallo-borospherene *D*_3h_ Ta_3_B_12_^–^ (**1**) has been very recently predicted by our group, which consists of two eclipsed B_3_ triangles on the top and bottom interconnected by three B_2_ units on the waist.^33^ However, there have been no experimental or theoretical evidence reported on tetra-Ta-doped boron clusters to date. Tetra-metal-doped endohedral metallo-silicon fullerenes *T*_d_ M_4_Si_28_ (M = Al and Ga) have been theoretically proposed to possess the same tetrahedral symmetry as their fullerene counterpart *T*_d_ C_28_.^34,35^ It is natural to ask at the current stage what GM structures the smallest tetra-Ta-doped boron fullerenes may have and if perfect spherical tetrahedral metallo-borospherenes are favored in thermodynamics over their alternative counterparts.

Based on extensive GM searches and first-principles theory calculations, we predict herein the perfect spherical tetrahedral metallo-borospherene *T*_d_ Ta_4_B_18_ (**2**), which possesses four equivalent B_3_ triangles at four corners interconnected by six B atoms on the edges, with four equivalent nonacoordinate Ta centers in four η^9^-B_9_ rings as integral parts of the cage surface, in the same tetrahedral symmetry as its fullerene counterpart *T*_d_ C_28_.^35^ The spherically aromatic Ta_4_B_18_ (**2**) possesses one 10c-2e B_9_(π)–Ta(d_σ_) bond and two 10c-2e B_9_(π)–Ta(d_δ_) coordination bonds over each Ta@B_9_ nonagon, with the remaining 18 valence electrons in nine 22c-2e bonds following the 18-electron rule (1S^2^1P^6^1D^10^). Ta_4_B_18_ (**2**) can be used as building blocks to form the face-centered three-dimensional (3D) Ta_4_B_15_ (**3**), which is metallic in nature.

## Results and Discussion

### Structures and Stabilities

**Figure 1:**
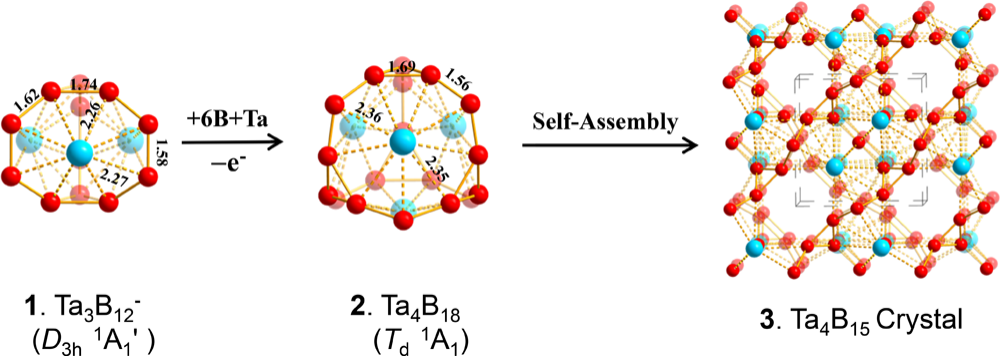
Optimized structures of Ta_3_B_12_^–^ (**1**), Ta_4_B_18_ (**2**), and 3D Ta_4_B_15_ crystal (**3**), with bond lengths indicated in Å in **1** and **2** at the PBE0 level.

**Figure 2:**
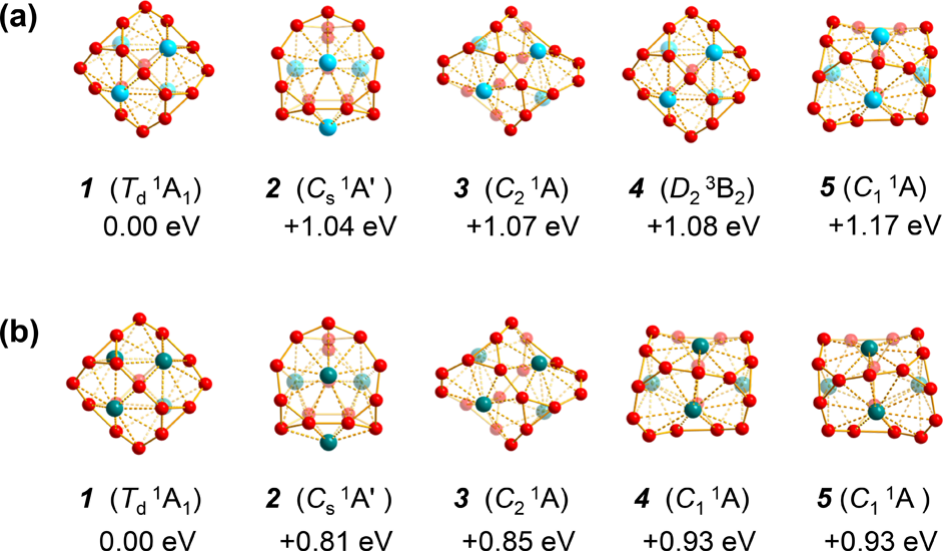
Five lowest-lying isomers of (a) Ta_4_B_18_ and (b) Nb_4_B_18_ with the relative energies indicated in eV at the CCSD(T) level.

Inspired by the newly reported smallest tri-metal-doped spherical trihedral metallo-borospherene *D*_3h_ Ta_3_B_12_^–^ (**1**),^33^ which contains two equivalent B_3_ triangles interconnected by three B_2_ units on the waist, with three octacoordinate Ta centers as integral parts of the cage surface, we manually designed the tetra-metal-doped perfect spherical tetrahedral metallo-borospherene *T*_d_ Ta_4_B_18_ (**2**) (^1^A_1_), which possesses four equivalent B_3_ triangles interconnected by six B atoms on the edges, with four nonacoordinate Ta centers in four η^9^-B_9_ rings as integrating parts of the cage surface (Figure 1). Interestingly and encouragingly, extensive TGMin GM searches^36,37^ show that Ta_4_B_18_ (**2**) is the well-defined GM of the neutral complex lying 1.04 eV lower than the second lowest-lying isomer *C*_s_ Ta_4_B_18_ (^1^A′) at the CCSD(T)^38−40^ level. All the other low-lying cage-like isomers appear to be at least 1.07 eV less stable than the GM in thermodynamics (Figure S1). The first slightly distorted triplet *D*_2_ Ta_4_B_18_ (^3^B_2_) is found to lie 1.08 eV higher than the *T*_d_ GM at CCSD(T). A similar situation exists in Nb_4_B_18_ for which the perfect tetrahedral *T*_d_ Nb_4_B_18_ also appears to be the well-defined GM of the neutral (Figure 2 and Figure S2). Ta_4_B_18_ (**2**) possesses the optimized B–B bond lengths of *r*_B–B_ = 1.56 between the B_3_ triangles at the corners and bridging B atoms on the edges, *r*′_B–B_ = 1.69 Å within the B_3_ triangles, and an average Ta–B coordination bond length of *r*_Ta–B_ = 2.35 Å between Ta atoms and their η^9^-B_9_ ligands. The large energy gaps between the highest occupied molecular orbitals (HOMOs) and lowest unoccupied molecular orbitals (LUMOs) of Δ*E*_gap_ = 2.63 and 2.60 eV calculated for *T*_d_ Ta_4_B_18_ and *T*_d_ Nb_4_B_18_, respectively, well support their high chemical stabilities, similar to the situations observed in cage-like *D*_2d_ B_40_ and *C*_3_/*C*_2_ B_39_^–^.^4,5^

Extensive molecular dynamics (MD) simulations indicate that Ta_4_B_18_ (**2**) is also highly dynamically stable, as evidenced by its small calculated average root-mean-square-deviation of RMSD = 0.12 Å and a maximum bond length deviation of MAXD = 0.41 Å at 1500 K (Figure S3a). Similarly, *T*_d_ Nb_4_B_18_ possesses a small calculated average root-mean-square-deviation of RMSD = 0.12 Å and a maximum bond length deviation of MAXD = 0.36 Å at 1200 K (Figure S3b). These highly stable tetra-Ta-doped spherical tetrahedral metallo-borospherenes possess the same tetrahedral symmetry as their carbon fullerene counterpart *T*_d_ C_28_.^35^*T*_d_ Ta_4_B_18_ (**2**) can be further self-assembled into the face-centered crystal Ta_4_B_15_ (**3**) (*P*-43*m*) in which each face-centering B atom is shared by two neighboring cubic unit cells, as shown in Figure 1. Ta_4_B_15_ (**3**) possesses the optimized lattice parameters of *a* = *b* = *c* = 5.77 Å at the PBE level,^42^ with both the B–B and B–Ta distances remaining basically unchanged compared to the corresponding bond length values in *T*_d_ Ta_4_B_18_ (**2**). The face-centering B atoms in Ta_4_B_15_ (**3**) are tetrahedrally coordinated, forming tetrahedral B(B)_4_ local structures with a B–B bond length of 1.57 Å. The calculated band structures of Ta_4_B_15_ (**3**) indicate that the face-centered 3D crystal is metallic in nature (Figure S4). Its projected density of states (PDOS) shows that both the B-2p orbitals and the Ta-5d orbitals contribute to the calculated PDOS near the Fermi level, with the former making major contributions to the PDOS above the Fermi level while the latter dominating the PDOS below the Fermi level.

### Natural Bonding Orbital and Bonding Pattern Analyses

The high stability of *T*_d_ Ta_4_B_18_ (**2**) originates from its unique structural and bonding patterns. Detailed natural bonding orbital (NBO) analyses show that the four equivalent nonacoordinate Ta atoms in Ta_4_B_18_ (**2**) possess the natural atomic charges of *q*_Ta_ = +0.94 |e|, electronic configurations of Ta[Xe]6s^0.23^5d^3.74^, total Wiberg bond indexes of WBI_Ta_ = 5.22, and Ta–B coordination bond orders of WBI_Ta–B_ = 0.41–0.45 (Ta–B interactions within the quasi-planar Ta@B_9_), indicating that each Ta atom donates its 6s^2^ electrons almost completely to the η^9^-B_9_ ligands while in return accepts roughly one electron (≈0.74 |e|) in its partially filled 5d orbitals from the surrounding B_9_ ligand via effective B(2p) → Ta(5d) backdonations. Such Ta–B coordination interactions appear to be comparable with those in the previously reported *D*_3h_ Ta_3_B_12_^–^, which has the Ta–B coordination bond order of WBI_Ta–B_ = 0.50–0.53 (Ta–B interactions within the Ta@B_8_ octagonal pyramids).^33^

**Figure 3:**
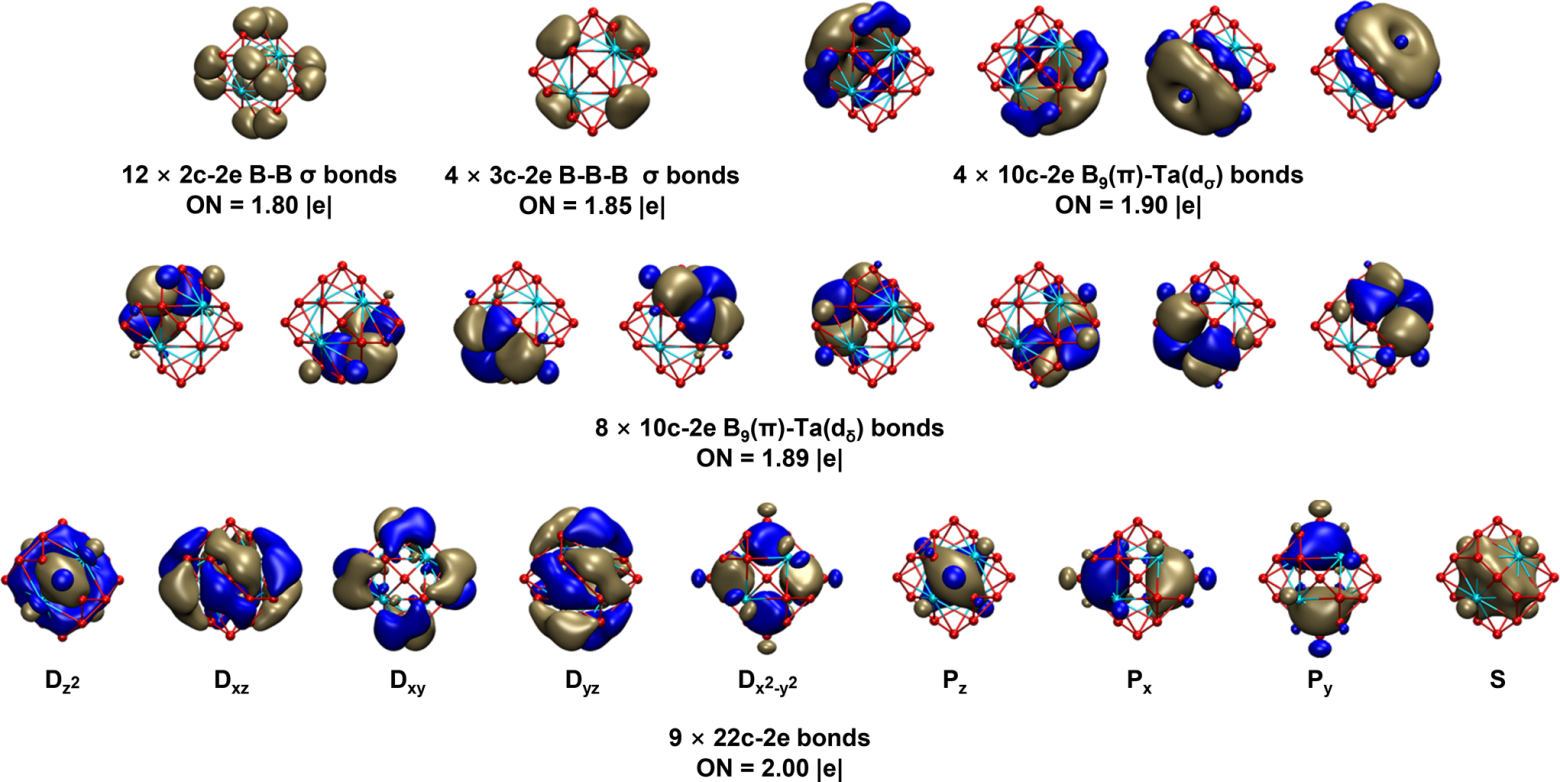
AdNDP bonding patterns of *T*_d_ Ta_4_B_18_ (**2**), with the occupation numbers (ONs) indicated.

Detailed adaptive natural density partitioning (AdNDP)^43,44^ bonding analyses shown in Figure 3 unveil both the localized and delocalized bonds of the system. *T*_d_ Ta_4_B_18_ (**2**) possesses 12 equivalent 2c-2e B–B σ bonds between four B_3_ triangles at the corners and six bridging B atoms on the edges and four equivalent 3c-2e σ bonds on four B_3_ triangles in the first row and 12 10c-2e B_9_–Ta coordination bonds evenly distributed on four equivalent Ta@B_9_ nonagonal faces, including four equivalent 10c-2e B_9_(π)–Ta(d_σ_) bonds in the first row and eight 10c-2e B_9_(π)–Ta(d_δ_) bonds in the second row. There exist thus one 10c-2e B_9_(π)–Ta(d_σ_) bond and two 10c-2e B_9_(π)–Ta(d_δ_) bonds over each Ta@B_9_ nonagon, forming a local 6-π aromatic system over each quasi-planar Ta@B_9_ unit to help stabilize the tetrahedral complex, similar to the situation in the experimentally observed aromatic inverse sandwich La_2_B_8_.^27^ The remaining 18 valence electrons occupy nine totally delocalized 22c-2e bonds in the third row, including one 22c-2e S-type bond, three 22c-2e P-type bonds, and five 22c-2e D-type bonds. The nine 22c-2e bonds well correspond to the superatomic electronic configuration (1S^2^1P^6^1D^10^) of *T*_d_ Ta_4_B_18_ (**2**) depicted in Figure S5. As shown in Figure S6, *T*_d_ Nb_4_B_18_ exhibits a similar bonding pattern. Such bonding patterns render spherical aromaticity to both *T*_d_ Ta_4_B_18_ (**2**) and *T*_d_ Nb_4_B_18_, as evidenced by the calculated large negative nucleus-independent chemical shift^45,46^ values of NICS = −141.9 and −124.9 ppm at their cage centers, respectively.

**Figure 4:**
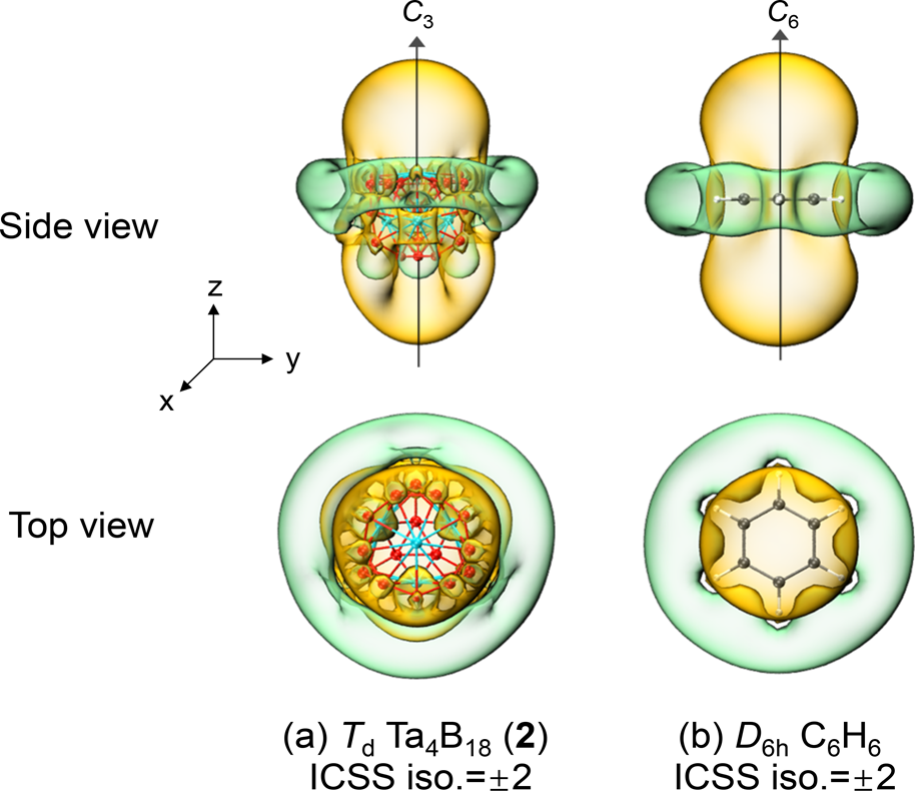
Comparison between the calculated iso-chemical shielding surfaces (ICSSs) of (a) Ta_4_B_18_ (**2**) and (b) *D*_6h_ C_6_H_6_, with the corresponding NICS-ZZ components indicated. The *C*_3_ axis of Ta_4_B_18_ (**2**) and the *C*_6_ axis of C_6_H_6_ are designated as the *z* axis in the vertical direction. Yellow regions stand for chemical shielding areas, while green areas represent chemical deshielding regions.

The spherical aromatic nature of *T*_d_ Ta_4_B_18_ (**2**) is further evidenced by its iso-chemical shielding surfaces (ICSSs)^47,48^ depicted in Figure 4a based on the calculated NICS-ZZ components, where the *z* axis is parallel to a *C*_3_ molecular axis of the system to show the chemical shielding around the Ta@B_9_ nonagon on the top, in comparison with the corresponding calculated ICSSs of benzene C_6_H_6_ in the *C*_6_ direction (Figure 4b). The areas highlighted in yellow inside the Ta_4_B_18_ tetrahedron and within about 1.0 A above the Ta centers in radial directions belong to chemical shielding regions with negative NICS-ZZ values, showing that the main aromatic contribution originates from Ta(5d)–B(2p) coordination interactions between the Ta centers and B_9_ ligands. The chemical deshielding areas with positive NICS-ZZ values highlighted in green are located outside the Ta@B_9_ nonagons in tangential directions. Interestingly, as clearly shown in Figure 4, the ICSSs of *T*_d_ Ta_4_B_18_ (**2**) appears to be similar to those of benzene *D*_6h_ C_6_H_6_ in radial directions, well demonstrating the aromatic nature of the spherical tetrahedral complex.

### Simulated IR, Raman, and UV–Vis Spectra

**Figure 5:**
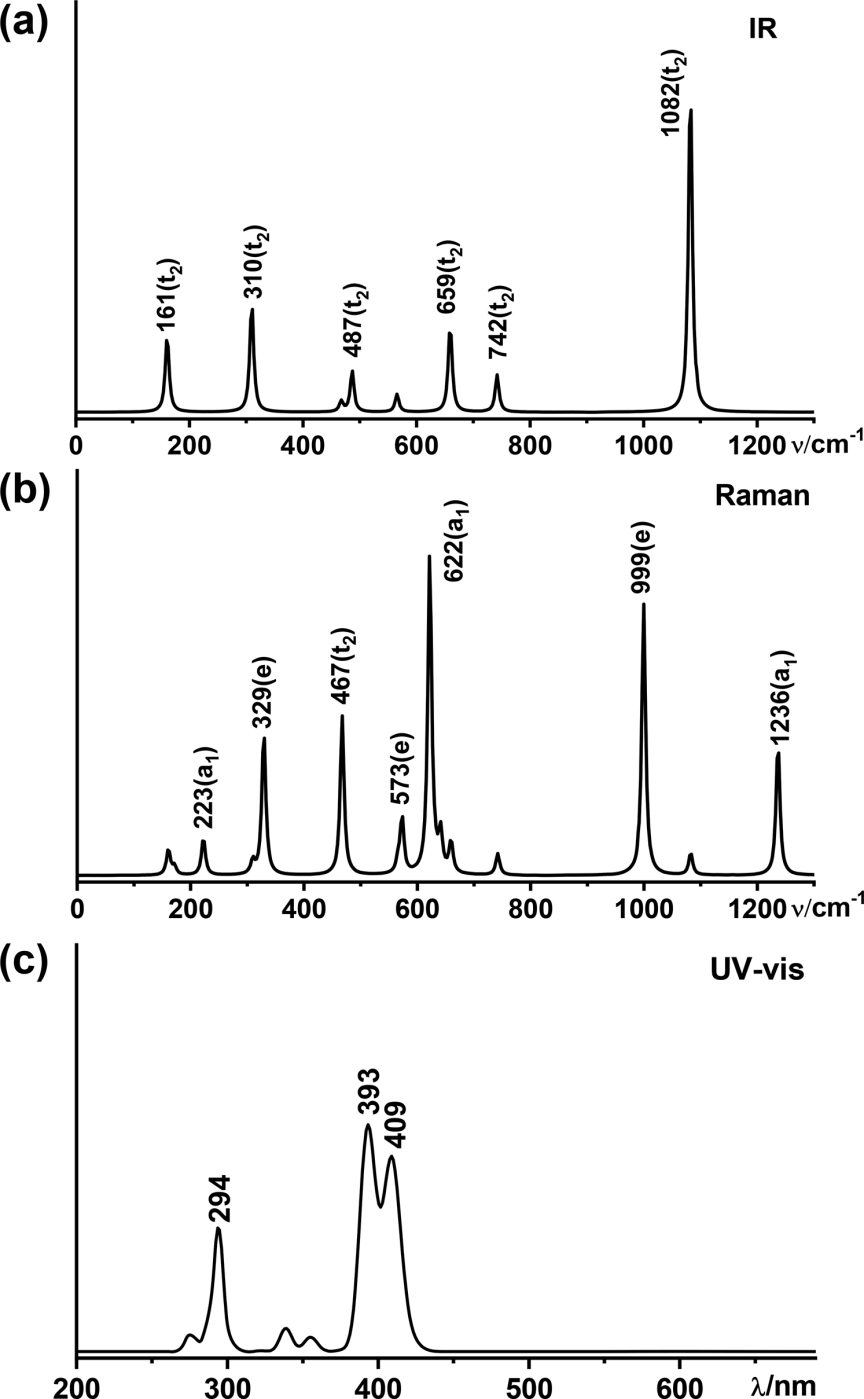
Simulated (a) IR, (b) Raman, and (c) UV–vis spectra of *T*_d_ Ta_4_B_18_ (**2**) at the PBE0 level.

The infrared (IR), Raman, and UV–vis spectra of *T*_d_ Ta_4_B_18_ (**2**) and *T*_d_ Nb_4_B_18_ are computationally simulated in Figure 5 and Figure S7 to facilitate their spectral characterizations. As shown in Figure 5, Ta_4_B_18_ (**2**) possesses four sharp IR peaks at 161(t_2_), 310(t_2_), 659(t_2_), and 1082(t_2_) cm^–1^ and five major Raman active vibrations at 329(e), 467(t_2_), 622(a_1_), 999(e), and 1236(a_1_) cm^–1^. The weak Raman peak at 223(a_1_) cm^–1^ and strongest Raman peak at 622(a_1_) cm^–1^ correspond to typical “radial breathing modes” (RBMs) of the cage-like structure, which can be used to characterize single-walled hollow boron nanostructures.^49^ The simulated UV–vis spectrum of Ta_4_B_18_ (**2**) with 150 excited states included in the calculations exhibits strong absorption peaks at 294, 393, and 409 nm, which mainly originate from electron transitions from the deep inner shells to the highly unoccupied molecular orbitals of Ta_4_B_18_ (**2**). *T*_d_ Nb_4_B_18_ exhibits similar spectral features to Ta_4_B_18_ (**2**) (Figure S7).

## Conclusions

Based on extensive GM searches and first-principles theory calculations, we have proposed in this work the perfect spherical tetrahedral metallo-borospherenes *T*_d_ Ta_4_B_18_ (**2**) and *T*_d_ Nb_4_B_18_, which possess the same tetrahedral symmetry as their carbon fullerene counterpart *T*_d_ C_28_. *T*_d_ M_4_B_18_ (M = Ta and Nb) appears to be spherically aromatic in nature, with 18 valence electrons occupying nine 22c-2e totally delocalized bonds following the superatomic electronic configuration of 1S^2^1P^6^1D^10^. Such highly stable spherically aromatic metallo-borospherene clusters may be synthesized and characterized in gas phases by laser ablations of Ta–B or Nb–B binary targets.^19−29,31^ As the smallest tetra-metal-doped metallo-borospherenes reported to date, they are possible to be self-assembled to form novel 3D boron nanostructures.

## Theoretical Procedure

Extensive GM searches on Ta_4_B_18_ and Nb_4_B_18_ were performed using the Tsinghua Global Minimum (TGMin) package,^36,37^ in conjunction with manual structural constructions based on the smallest metallo-borospherene *D*_3h_ Ta_3_B_12_^–^ (**1**).^33^ More than 1500 singlet or triplet stationary points were explored for each cluster at the PBE/DZVP level.^42^ Low-lying isomers were then fully optimized at the PBE0^41^ level with the 6-31+G* basis set^50^ for B and the Stuttgart (2f1g) pseudopotential^51,52^ for Ta and Nb using the Gaussian-16 program suite,^53^ with vibrational frequencies checked to make sure that all isomers reported are true minima. The 10 lowest-lying isomers were subsequently reoptimized using the PBE0 functional with the aug-cc-pVTZ basis set^54,55^ for B and the Stuttgart (2f1g) pseudopotential for Ta and Nb. Relative energies for the five lowest-lying isomers were further refined for Ta_4_B_18_ using the more accurate coupled cluster method with triple excitations CCSD(T)^38−40^ implemented in Molpro^56^ with the basis set of 6-31G(d) for B and the Stuttgart (2f1g) pseudopotential for Ta. The calculations on the 3D Ta_3_B_15_ crystal (**3**) were performed using the Vienna ab initio simulation package (VASP)^57,58^ within the framework of the projector-augmented wave (PAW) pseudopotential method^59,60^ and PBE generalized gradient approximation (GGA).^42,61^ Natural bonding orbital analyses were performed using the NBO 6.0 program.^62^ Nucleus-independent chemical shifts (NICS)^45,46^ were calculated at the cage centers to assess the spherical aromaticity of tetrahedral metallo-borospherenes. The iso-chemical shielding surfaces (ICSSs)^47,48^ were generated with the Multiwfn 3.7 code.^63^ Chemical bonding was analyzed using the adaptive natural density partitioning (AdNDP) method,^43,43^ which has been successfully applied to various organic and inorganic species.^2−10,18,19^ Molecular dynamics (MD) simulations were carried out on *T*_d_ Ta_4_B_18_ for 30 ps using a CP2K software suite.^64^ The iso-surface maps of various orbitals and the iso-chemical shielding surfaces (ICSSs) were realized using the visual molecular dynamics (VMD) software.^65^

## Supporting Information Available

Low-lying isomers of Ta_4_B_18_ at the PBE0 and CCSD(T) levels (Figure S1); low-lying isomers of Nb_4_B_18_ at the PBE0 and CCSD(T) levels (Figure S2); MD simulations of Ta_4_B_18_ (**2**) at 1500 K and *T*_d_ Nb_4_B_18_ at 1200 K (Figure S3); calculated band structures and projected densities of states (PDOS) of Ta_4_B_15_ (**3**) at the PBE level (Figure S4); the superatomic electronic configuration (1S^2^1P^6^1D^10^) of *T*_d_ Ta_4_B_18_ (**2**) (Figure S5); AdNDP bonding patterns of *T*_d_ Nb_4_B_18_ (Figure S6); simulated IR, Raman, and UV–vis spectra of *T*_d_ Nb_4_B_18_ at the PBE0 level (Figure S7); optimized coordinates of *T*_d_ Ta_4_B_18_ (**2**) and *T*_d_ Nb_4_B_18_ at PBE0 (Table S1); and optimized coordinates (*x*, *y*, *z*) of the 3D Ta_4_B_15_ (**3**) crystal at the PBE level (Table S2) (PDF)
