# Supplementary material for: Perfect Spherical Tetrahedral Metallo-Borospherene Ta4B18 as a Superatom Following the 18-Electron Rule
Source: ACS Omega. 2021 Apr 12;6(16):10991–6. doi: 10.1021/acsomega.1c00828 (PMC8153900; doi:10.1021/acsomega.1c00828)
Supplement: Supplementary file 1 — ao1c00828_si_001.pdf [file ao1c00828_si_001.pdf]

Supporting Information

Perfect Spherical tetrahedral Metallo-Borospherene Ta_4B_{18} As a Superatom Following the 18-electron Rule

Yu Zhang, Xiao-Qin Lu, Miao Yan, and Si-Dian Li*

Institute of Molecular Science, Shanxi University, Taiyuan, 030006, China.

Contents

Figure S1. Low−lying isomers of Ta_4B_{18} at PBE0 and CCSD(T) levels.

Figure S2. Low−lying isomers of Nb_4B_{18} at PBE0 and CCSD(T) levels.

Figure S3. MD simulations of (a) Ta_4B_{18} (**2**) at 1500 K and (b) T_d Nb_4B_{18} at 1200 K.

Figure S4. Calculated band structures and projected densities of states (PDOS) of 3D Ta_4B_{15} (**3**) at PBE level.

Figure S5. The superatomic electronic configuration ($1S^21P^61D^{10}$) of T_d Ta_4B_{18} (**2**)

Figure S6. AdNDP bonding patterns of T_d Nb_4B_{18}.

Figure S7. Simulated (a) IR, (b) Raman, and (c) UV-vis spectra of T_d Nb_4B_{18} at PBE0 level.

Table S1. Optimized coordinates of (a) T_d Ta_4B_{18} (**2**), (b) T_d Nb_4B_{18} at PBE0.

Table S2. Optimized coordinates (x, y, z) of the 3D Ta_4B_{15}(**3**) crystal at PBE level.

Figure S1. Low−lying isomers of Ta_4B_{18} (***1***-***12***) at PBE0/B/6-31+G(d)/Ta/Stuttgart(2f1g), PBE0//B/aug-cc-pVTZ/Ta/Stuttgart(2f1g) (in parentheses), and CCSD(T)/B/6-31G(d)/Ta/Stuttgart(2f1g) (in square brackets) levels, with the relative energies indicated in eV.

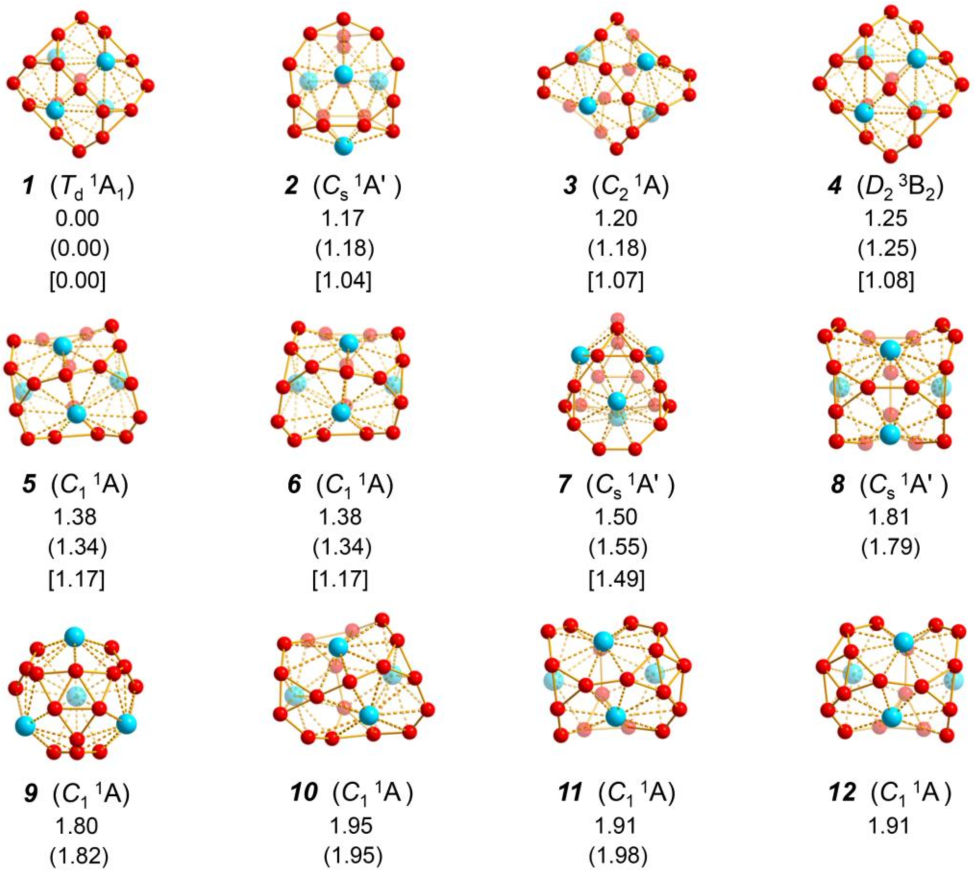

Figure S2. Low−lying isomers of Nb_4B_{18} (***1***-***12***) at PBE0/B/6-31+G(d)/Nb/Stuttgart(2f1g), PBE0//B/aug-cc-pVTZ/Nb/Stuttgart(2f1g) (in parentheses), and CCSD(T)/B/6-31G(d)/Nb/Stuttgart(2f1g) (in square brackets) levels, with the relative energies indicated in eV.

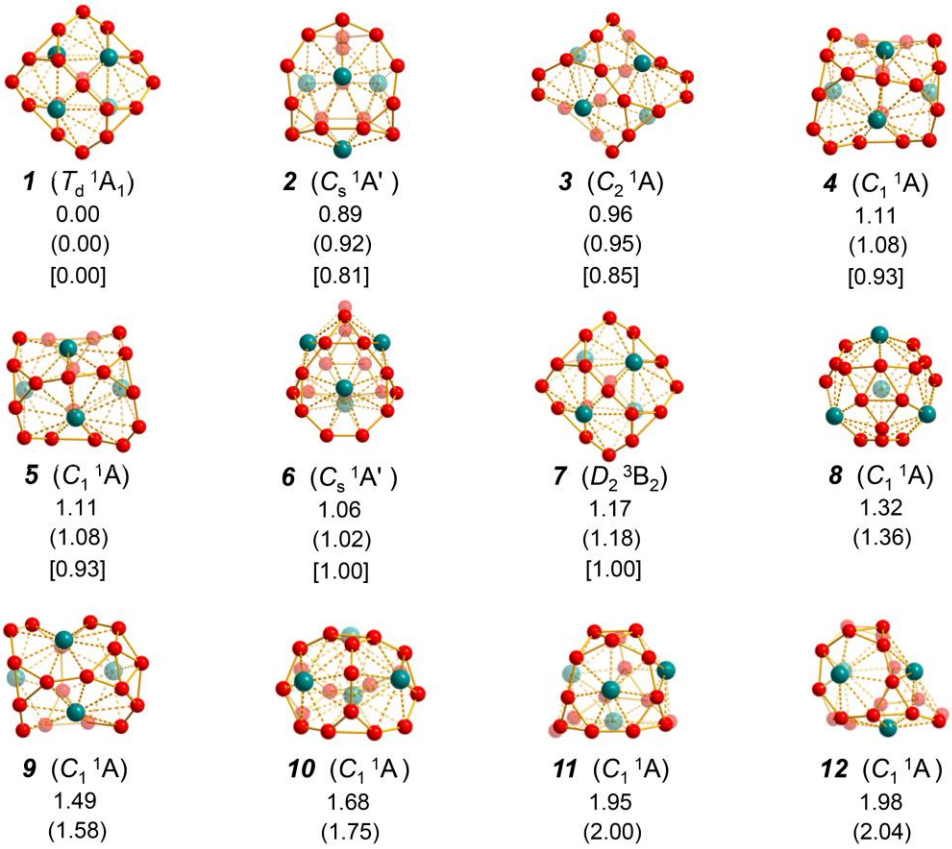

Figure S3. MD simulations of (a) Ta_4B_{18} (**2**) at 1500 K and (b) T_d Nb_4B_{18} at 1200 K, respectively, with the calculated average root−mean−square−deviations (RMSD) and maximum bond length deviations (MAXD) indicated.

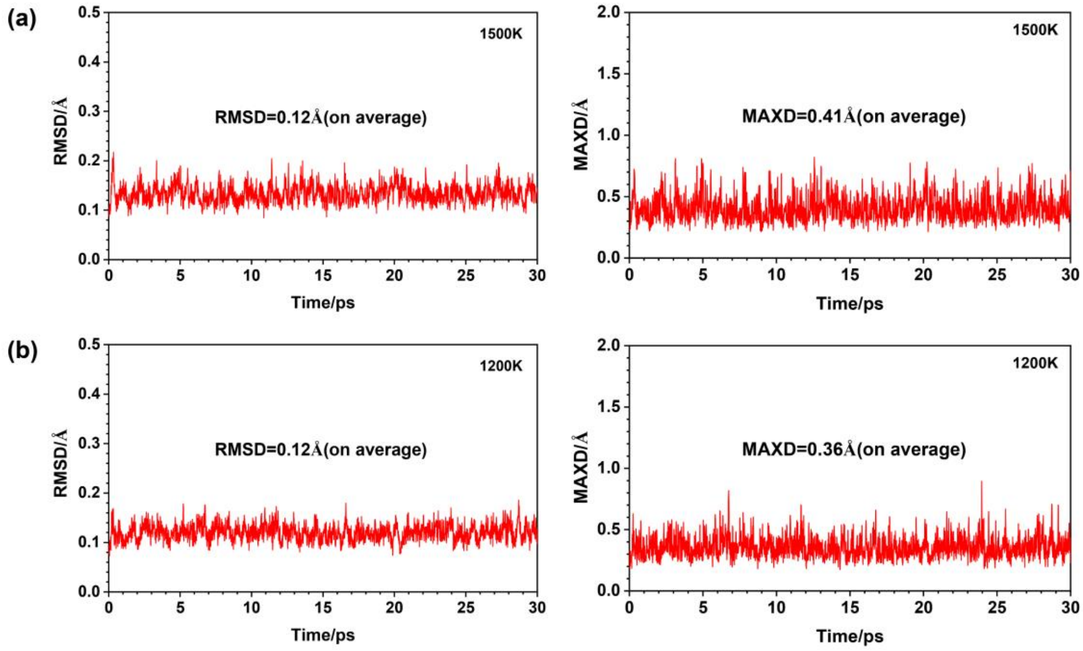

Figure S4. Calculated band structures and projected densities of states (PDOS) of Ta_4B_{15} (**3**) at PBE level.

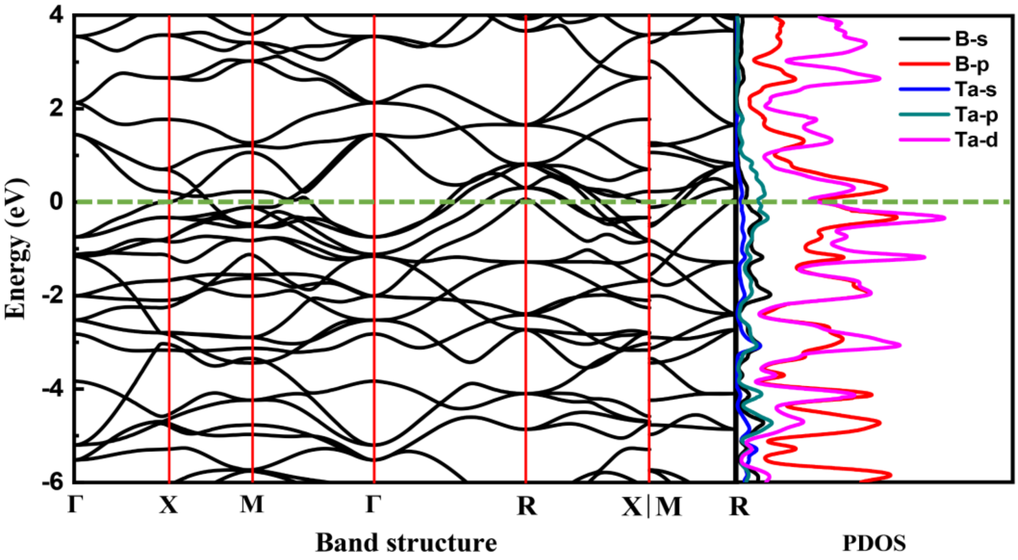

Figure S5. The superatomic electronic configuration ($1S^21P^61D^{10}$) of T_d Ta_4B_{18} (**2**) at PBE0//B/aug-cc-pVTZ/Ta/Stuttgart(2f1g)level.

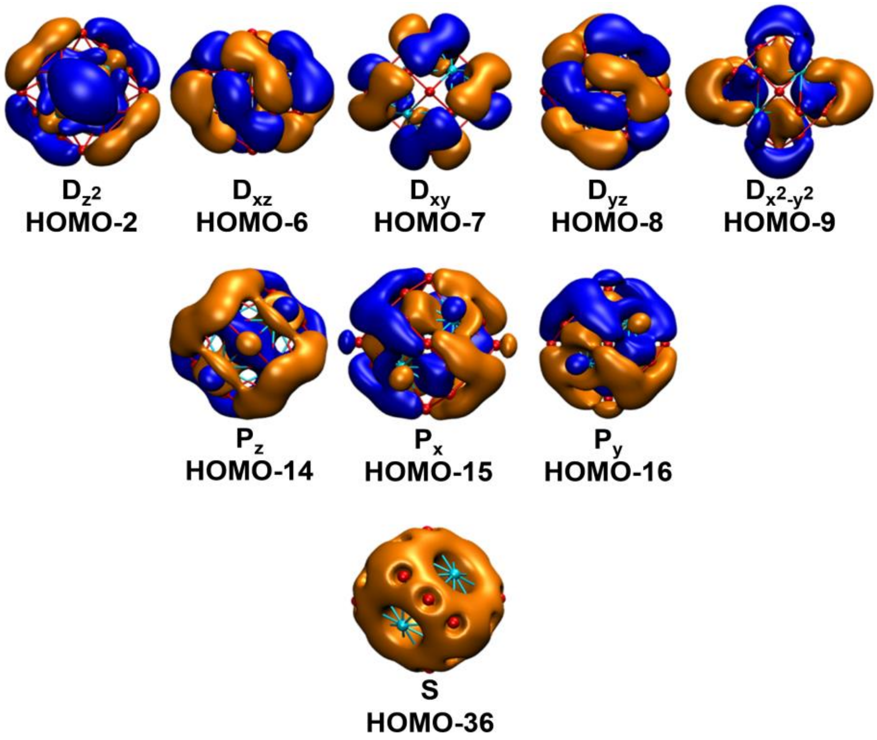

Figure S6. AdNDP bonding patterns of T_d Nb_4B_{18}, with the occupation numbers (ON) indicated.

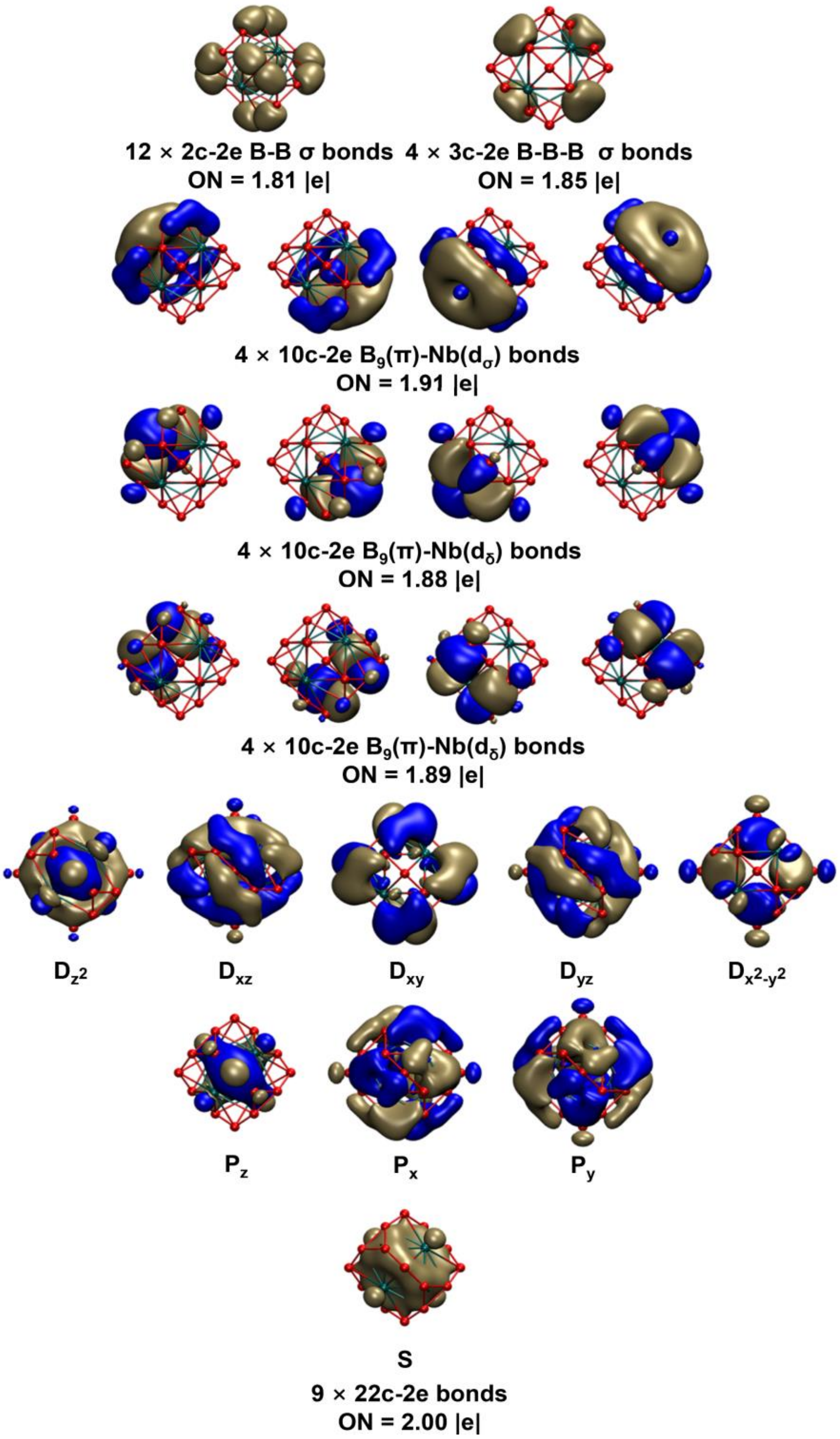

Figure S7. Simulated (a) IR, (b) Raman, and (c) UV-vis spectra of T_d Nb_4B_{18} at PBE0//B/6-31+G*/Nb/Stuttgart(2f1g).

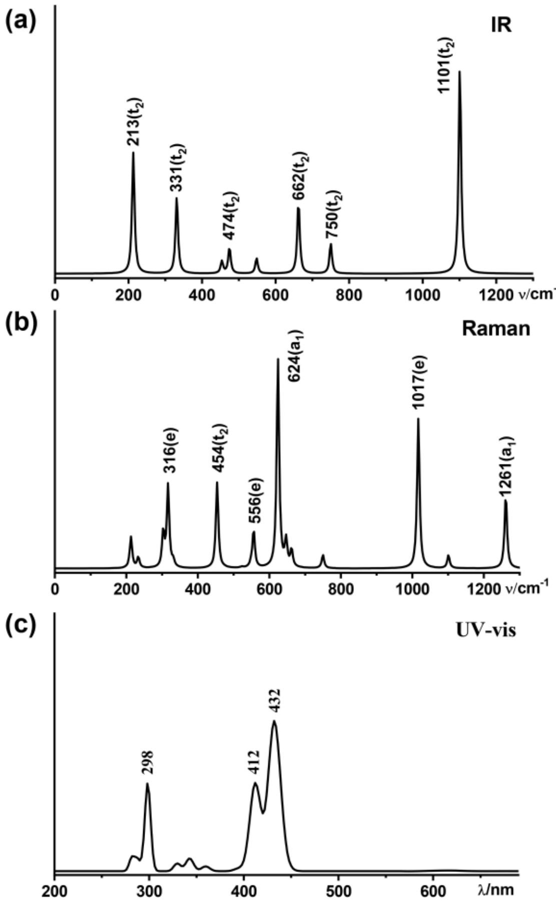

Table S1. Optimized coordinates of (a) T_d Ta_4B_{18} (**2**) at PBE0//B/aug-cc-pVTZ/Ta/Stuttgart(2f1g) and (b) T_d Nb_4B_{18} at PBE0//B/aug-cc-pVTZ/Nb/Stuttgart(2f1g).

(a) T_d Ta_4B_{18} (**2**)

B	0.00000000	0.00000000	-2.87740000
B	0.00000000	2.87740000	0.00000000
B	-2.87740000	0.00000000	0.00000000
B	0.99427500	2.19213500	0.99427500
B	0.99427500	0.99427500	2.19213500
B	0.00000000	-2.87740000	0.00000000
B	-0.99427500	-2.19213500	0.99427500
B	0.00000000	0.00000000	2.87740000
B	0.99427500	-0.99427500	-2.19213500
B	0.99427500	-2.19213500	-0.99427500
B	2.87740000	0.00000000	0.00000000
B	2.19213500	-0.99427500	-0.99427500
B	2.19213500	0.99427500	0.99427500
B	-2.19213500	-0.99427500	0.99427500
B	-0.99427500	-0.99427500	2.19213500
Ta	1.06480200	-1.06480200	1.06480200
Ta	-1.06480200	1.06480200	1.06480200
Ta	-1.06480200	-1.06480200	-1.06480200
B	-0.99427500	2.19213500	-0.99427500
B	-2.19213500	0.99427500	-0.99427500
Ta	1.06480200	1.06480200	-1.06480200
B	-0.99427500	0.99427500	-2.19213500

(b) T_d Nb_4B_{18}

B	0.00000000	2.87916900	0.00000000
B	2.87916900	0.00000000	0.00000000
B	0.00000000	0.00000000	-2.87916900
B	2.19003700	-0.99468300	0.99468300
B	0.99468300	-2.19003700	0.99468300
B	-2.87916900	0.00000000	0.00000000
B	-2.19003700	-0.99468300	-0.99468300
B	0.00000000	-2.87916900	0.00000000
B	-0.99468300	2.19003700	0.99468300
B	-2.19003700	0.99468300	0.99468300
B	0.00000000	0.00000000	2.87916900
B	-0.99468300	0.99468300	2.19003700
B	0.99468300	-0.99468300	2.19003700
B	-0.99468300	-0.99468300	-2.19003700
B	-0.99468300	-2.19003700	-0.99468300

B	2.19003700	0.99468300	-0.99468300
B	0.99468300	0.99468300	-2.19003700
B	0.99468300	2.19003700	-0.99468300
Nb	1.06408300	1.06408300	1.06408300
Nb	-1.06408300	-1.06408300	1.06408300
Nb	1.06408300	-1.06408300	-1.06408300
Nb	-1.06408300	1.06408300	-1.06408300

Table S2. Optimized coordinates (x, y, z) of Ta_4B_{15} (**3**) crystal at PBE level

B	0.119450003	0.327250004	0.672749996
B	0.880550027	0.672749996	0.672749996
B	0.880550027	0.327250004	0.327250004
B	0.119450003	0.672749996	0.327250004
B	0.672749996	0.119450003	0.327250004
B	0.672749996	0.880550027	0.672749996
B	0.327250004	0.880550027	0.327250004
B	0.327250004	0.119450003	0.672749996
B	0.327250004	0.672749996	0.119450003
B	0.672749996	0.672749996	0.880550027
B	0.327250004	0.327250004	0.880550027
B	0.672749996	0.327250004	0.119450003
B	0.500000000	0.000000000	0.500000000
B	0.500000000	0.500000000	0.000000000
B	0.000000000	0.500000000	0.500000000
Ta	0.315549999	0.684449971	0.684449971
Ta	0.684450030	0.315550029	0.684449971
Ta	0.684450030	0.684449971	0.315550029
Ta	0.315549999	0.315550029	0.315550029
